# Mystifying lesions in syphilis-HIV co-infection

**DOI:** 10.1186/1471-2334-14-S7-P45

**Published:** 2014-10-15

**Authors:** Anca Raducan, Adina Alexandru, Doina Stefan, Catalina Stoian, Rodica Olteanu

**Affiliations:** 1 Second Dermatology Clinic, Colentina Clinical Hospital, Bucharest, Romania; 2 Dermatology Department, Carol Davila University of Medicine and Pharmacy, Bucharest, Romania

## Background

Syphilis-HIV co-infection is common in seropositive patients and it is important to screen for both diseases, especially in patients with behavioral risk.

## Case report

We report the case of a 22 years old male patient, who presented with a vesicular eruption, bacterial over infected, with a group pattern distribution throughout the entire skin, evolving for approximately three months. The patient was previously treated for this condition with systemic antibiotics, which he could not mention, being diagnosed with superficial folliculitis. However, no improvement was noticed. He denied unprotected sexual intercourse. The patient was clinically examined and we performed routine tests, immunologic assays, bacteriological swab and skin biopsy.

General examination showed generalized, painless micropoly-lymphadenopathy and continuous muscle pain in the lower legs. Dermatological assessment of lesions revealed a disseminated eruption on the entire skin, including the face and penis sheath, consisting of erythematous plaques with the surface covered by crusts and vesicles arranged in groups, with various sizes (diameter 1-3 cm) and clearly defined edges. The presumptive diagnosis was secondary syphilis, and in order to rule out the differential diagnosis of pityriasis lichenoides et varioliformis acuta, eosinophilic folliculitis and systemic lupus erythematosus we performed a series of tests including immunologic assays, viral markers for HCV, serology for syphilis, bacteriological swab from the lesions and a skin biopsy. The results were positive for syphilis and *Staphylococcus aureus* was isolated from the lesions. Histopathological appearance of psoriasiform and lichenoid dermatitis with plasma cells was interpreted in the context of secondary syphilis, confirming our diagnosis. The patient eventually admitted having multiple unprotected sexual contacts. We tested for HIV Ag/Ac (1+2), which was positive.

## Conclusion

The patient was treated for syphilis in our department and he was referred to the infectious diseases hospital for proper evaluation and treatment for HIV infection. The particularity of the case consists in the uncommon aspect of the eruption in this HIV-syphilis co-infection.

## Consent

Written informed consent was obtained from the patient for publication of this Case report and any accompanying images. A copy of the written consent is available for review by the Editor of this journal.

